# Microfluidic Isolation of Circulating Tumor Cell Clusters by Size and Asymmetry

**DOI:** 10.1038/s41598-017-01150-3

**Published:** 2017-05-26

**Authors:** Sam H. Au, Jon Edd, Amy E. Stoddard, Keith H. K. Wong, Fabio Fachin, Shyamala Maheswaran, Daniel A. Haber, Shannon L. Stott, Ravi Kapur, Mehmet Toner

**Affiliations:** 1 Center for Engineering in Medicine, Massachusetts General Hospital, Harvard Medical School, Boston, MA 02114 USA; 2 Department of Surgery, Massachusetts General Hospital, Harvard Medical School, Boston, MA 02114 USA; 3 Massachusetts General Hospital Center for Cancer Research, Harvard Medical School, Charlestown, MA 02129 USA; 4 Department of Medicine, Massachusetts General Hospital, Harvard Medical School, Boston, MA 02114 USA; 5 Howard Hughes Medical Institute, Bethesda, MD 20815 USA; 6 Shriners Hospital for Children, Boston, MA 02114 USA

## Abstract

Circulating tumor cell clusters (CTC clusters) are potent initiators of metastasis and potentially useful clinical markers for patients with cancer. Although there are numerous devices developed to isolate individual circulating tumor cells from blood, these devices are ineffective at capturing CTC clusters, incapable of separating clusters from single cells and/or cause cluster damage or dissociation during processing. The only device currently able to specifically isolate CTC clusters from single CTCs and blood cells relies on the batch immobilization of clusters onto micropillars which necessitates long residence times and causes damage to clusters during release. Here, we present a two-stage continuous microfluidic chip that isolates and recovers viable CTC clusters from blood. This approach uses deterministic lateral displacement to sort clusters by capitalizing on two geometric properties: size and asymmetry. Cultured breast cancer CTC clusters containing between 2–100 + cells were recovered from whole blood using this integrated two-stage device with minimal cluster dissociation, 99% recovery of large clusters, cell viabilities over 87% and greater than five-log depletion of red blood cells. This continuous-flow cluster chip will enable further studies examining CTC clusters in research and clinical applications.

## Introduction

The isolation of circulating tumor cells (CTCs) from patient blood specimens has the potential to significantly improve cancer treatment by revealing information that may enable more accurate prognoses, better predictions of how tumors will respond to therapeutic options, and a more comprehensive understanding of the cellular mechanisms involved in metastasis. The many potential uses of CTCs have led to the development of large numbers of technologies to capture these rare cellular events from blood^1, 2^.

Individual CTCs however, are not the only valuable cells for isolation from patient blood. Multicellular aggregates of CTCs (CTC clusters) may be 100-fold more metastasis competent than single CTCs^3–5^. In mouse models, CTC clusters are responsible for seeding half or more of secondary metastatic tumors^3, 4^ and in humans, the presence of even a single CTC cluster in sampled blood is correlated with significantly reduced progression free survival rates in patients with prostate^4^, breast^4^ and small-cell lung^6^ cancers. The numerous devices that have been developed for the capture of individual CTCs are only rarely capable of capturing these clusters intact. Given the large variability in reported cluster capture efficiencies and the large shear rates inherent in many devices, it is likely that the majority of CTC clusters in sampled patient blood escape capture or dissociate into single cells or smaller clusters during processing^7^.

We have previously reported a technology designed specifically to capture CTC clusters. This technology uses triangular micropillars to immobilize CTC clusters from whole blood^7^. Although this device is capable of high efficiency physical cluster capture, it has two drawbacks that limit its ability to provide viable CTC clusters for downstream analysis. First, the physical capture mechanism relies on batch processing which, results in long on-chip residence times during which CTC clusters may be caught within micropillars experiencing blood shear flow for hours. Second, elevated shear stresses are needed to release the majority of clusters from micropillars^7^. Both of these drawbacks do not prevent the detection of CTC clusters, but limit the applicability of released clusters because of potential changes or damage to cellular structures and/or content.

To enable the recovery CTC clusters for sensitive functional analyses, we present a continuous flow microfluidic device for isolating intact CTC clusters from whole blood. The device uses a two-stage deterministic lateral displacement (DLD) strategy^8–10^. The first stage is a ‘standard’ DLD device designed to extract large clusters based on size. The second stage, whose input is the undeflected product of Stage 1, uses asymmetrical pillars and height restrictions to extract smaller clusters based on the inherent asymmetric nature of multicellular aggregates. This next generation cluster chip isolates high integrity CTC clusters from whole blood that experience physiological-or-lower shear stress rates throughout, on-chip residence times on the order of seconds which minimizes damage or processing bias, and the continuous-flow nature of the chip enables in-line integration with other analysis and capture strategies.

## Results

### Cluster Capture Principles

**Figure 1:**
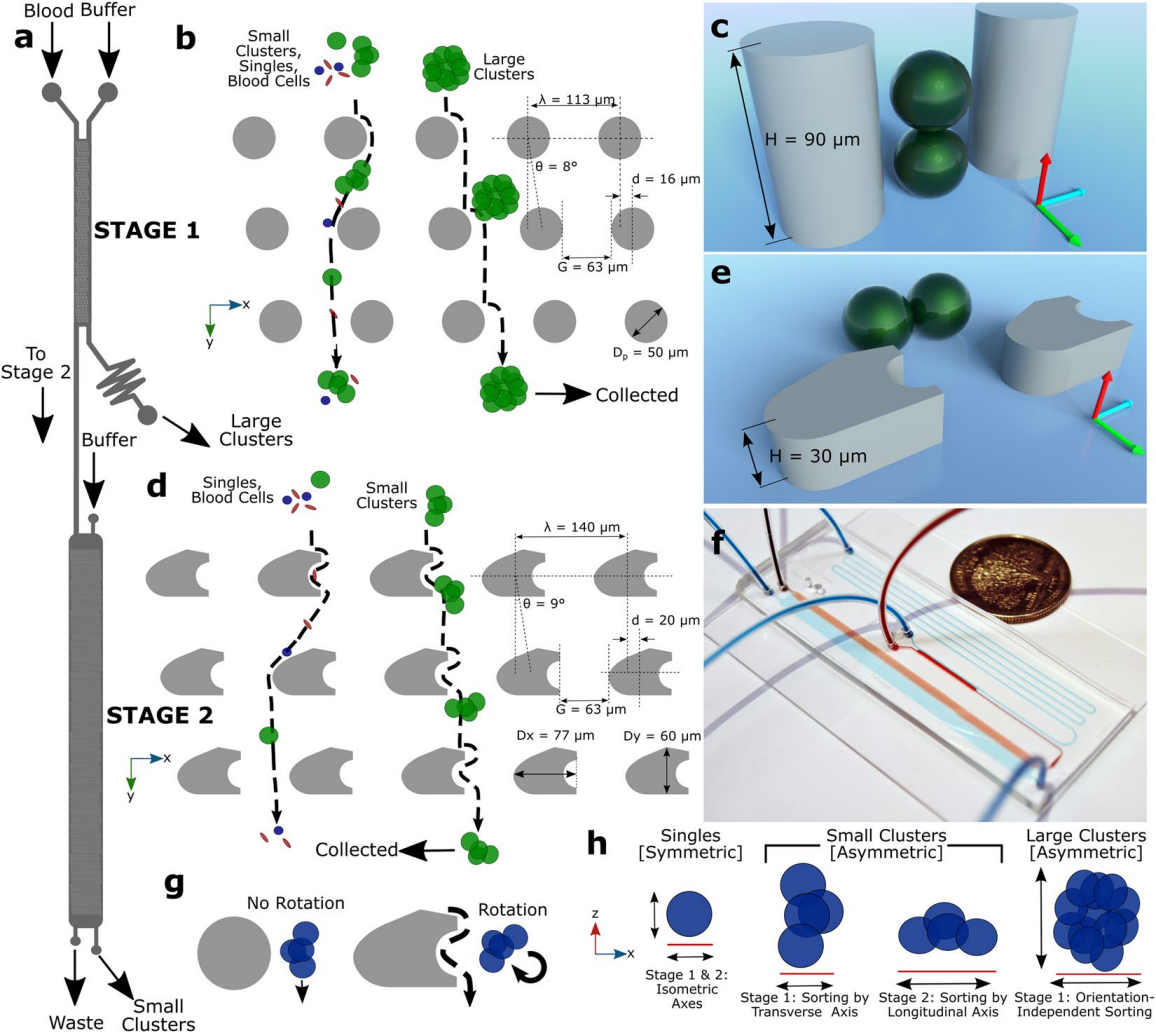
Two-Stage Cluster Capture Operating Principles: (**a**) Stage 1 and Stage 2 device inlet, outlets and fluidic paths. (**b**) Array of cylindrical micropillars in Stage 1 deflects large clusters from other blood cells using deterministic lateral displacement. (**c**) A 90 µm Stage 1 ceiling allows clusters to align along the Z-axis. (**d**) Array of asymmetric pillars in Stage 2 deflects small clusters. (**e**) A 30 µm Stage 2 ceiling forces clusters to align in the X-Y plane. (**f**) Photograph of device running whole blood (red) with colored PBS buffer (blue) next to U.S. quarter dollar coin. (**g**) Diagram showing asymmetric pillars inducing cluster rotation. (**h**) Symmetric single cells do not deflect while asymmetric small clusters sort by their longitudinal axes in Stage 2 and large clusters sort by size in Stage 1. Schematics not to scale.

We developed an integrated two-stage strategy for capturing CTC clusters that relies on the combination of two characteristics of CTC clusters, that when probed together, can be used to effectively distinguish them from other cells in whole blood: size and asymmetry (Fig. 1).

The first stage of the microfluidic device, “Stage 1”, was designed to remove large clusters from whole blood (Fig. 1a) by deterministic lateral displacement (DLD), which isolates large clusters based on their greater sizes. Stage 1 consists of an array of 50 µm diameter, 90 µm high polydimethylsiloxane cylindrical micropillars with 63 µm gaps between pillars and row shift ratios of 1/7 (Fig. 1b). These geometries were designed to deflect particles with 30 µm or greater effective diameters while permitting very large clusters, such as those containing over 100 cells^11^, to pass without clogging. Because of the high ceiling in Stage 1, blood cells^12, 13^ and small clusters tend to align their longest (longitudinal) axes vertically in the z-direction (Fig. 1c) or other similar alignments that result in sorting based on shorter (transverse) cluster axes^13^ (Movie S1). Thus, the sized-based sorting in Stage 1 was designed to deflect CTC clusters of large size for which orientation is unimportant since their shortest axial diameters are 30 µm or greater. Successfully deflected clusters are collected in the Stage 1 product stream, while remaining clusters, cancer cells and blood are shuttled into the inlet of the second device stage (Stage 2).

Stage 2 was designed to sort clusters that failed to sort in Stage 1 by discriminating asymmetric clusters from symmetric single cells. Stage 2 uses a DLD array of micropillars with 63 µm gaps between pillars, row shift ratios of 1/7 to displace clusters (Fig. 1d), similar to the first stage. However, two key geometric features are different in Stage 2. First, this stage has a ceiling of 30 µm which constrains the majority of clusters to align their longitudinal axes “flat” in the X-Y plane (Fig. 1e). Height restrictions of this sort have been previously used to force red blood cell to align so that their long ~8 µm axes can be used for DLD sorting^12, 13^. Second, the pillars in Stage 2 were designed as asymmetric hybrids of “I”-shaped pillars and elliptic cylinders (Fig. 1d,g). The “I”-shaped grooves in pillars cause disturbances to typically smooth streamlines which induces rotation in asymmetrical particles^14^ such as clusters (Fig. 1g); while ellipsoidal pillar asymmetries inhibit streamline symmetry between pillars. Because of their complex geometries, the precise critical diameter in Stage 2 arrays is difficult to determine. Therefore, pillars were approximated as rectangular prisms that provided critical diameters of approximately 30 µm. The ability of Stage 2 pillars to induce rotational forces and disrupt streamline symmetry between pillars was validated using 3D computational fluid dynamics simulations (Fig. S1) and their ability to induce cluster rotation leading to deflection of small clusters was visually verified (Fig. S2 and Movie S2). Furthermore, the ability of asymmetric pillars to promote the capture of small CTC clusters in buffer was experimentally evaluated using 30 µm high prototype devices used for development (n = 3). Asymmetric pillars led to a 64% increase in the capture efficiencies of small clusters when compared to standard cylindrical pillars (69 ± 10% vs. 42 ± 12%). Thus, these two design considerations, restricted device ceilings and asymmetric pillars, force clusters to align during deterministic displacement in manners that typical DLD devices are incapable of achieving (Movie S3). Since single cells in suspension have spherical symmetry, these cells remain undeflected (Fig. 1h). To achieve perfect deflection, small clusters in Stage 2 would need to align their longitudinal axes perpendicular to the flow direction between every post (Fig. 1e). In reality, clusters assumed a continuum of alignments within the X-Y plane (Fig. 1g). Although not optimal, the majority of these alignments still enabled correct DLD deflection for small clusters (Movies S2, S5 and S6). Nonetheless, to account for deflection failures, an extra 16 resets (beyond the theoretical minimum of 32) were built into DLD arrays to provide a significant margin of safety. Furthermore, devices consisting of only Stage 2 geometries could theoretically be used without the first stage, but the low device heights required for Stage 2 operation leads to device clogging and disruption of large clusters when operated without the first stage (data not shown). Thus, this integrated two-stage strategy gently isolates intact clusters based on size in Stage 1 and asymmetry in Stage 2 (Fig. 1h).

### Computational Fluid Dynamics

Three dimensional steady-state fluid dynamic simulations of Stage 1 and Stage 2 geometries were performed using ANSYS Fluent (Fig. S1). Mid-plane fluid streamlines were computed to evaluate the ability of asymmetrical pillar geometries to disturb fluid symmetry and promote rotation in comparison to standard cylindrical pillars. While fluid streamlines between typical (Stage 1) cylindrical pillars were highly symmetrical (Fig. S1a), asymmetrical pillars used in Stage 2 generated significant disturbance and asymmetry into the streamlines between pillars (Fig. S1b). The pillar geometry chosen for Stage 2 was chosen because this geometry demonstrated a high degree of asymmetry and streamline disturbance. Furthermore, fluid streamlines were used to validate that the critical diameter of DLD arrays in Stage 2 was approximately 30 µm as designed, by doubling the width of the first streamline from each post and averaging for all rows within each reset (Fig. S1)^10^.

To determine flow rate limits that minimized cell damage, peak shear stresses (which occurred at the narrowest segments between pillars) were determined for a range of cluster processing rates. A cell/blood flow rate of 0.5 mL/hr generated a peak shear stress of 2.9 Pa in Stage 1 (Fig. S1c) and 4.8 Pa in Stage 2 (Fig. S1d). These values were less than the peak arterial shear stress experienced by cells in normal human vasculature of 5–20 Pa^15^ and therefore this rate was chosen for the majority of characterization studies described below since clusters should be resilient to these stresses during the short on-chip residence times.

### Isolation of Clusters

**Figure 2:**
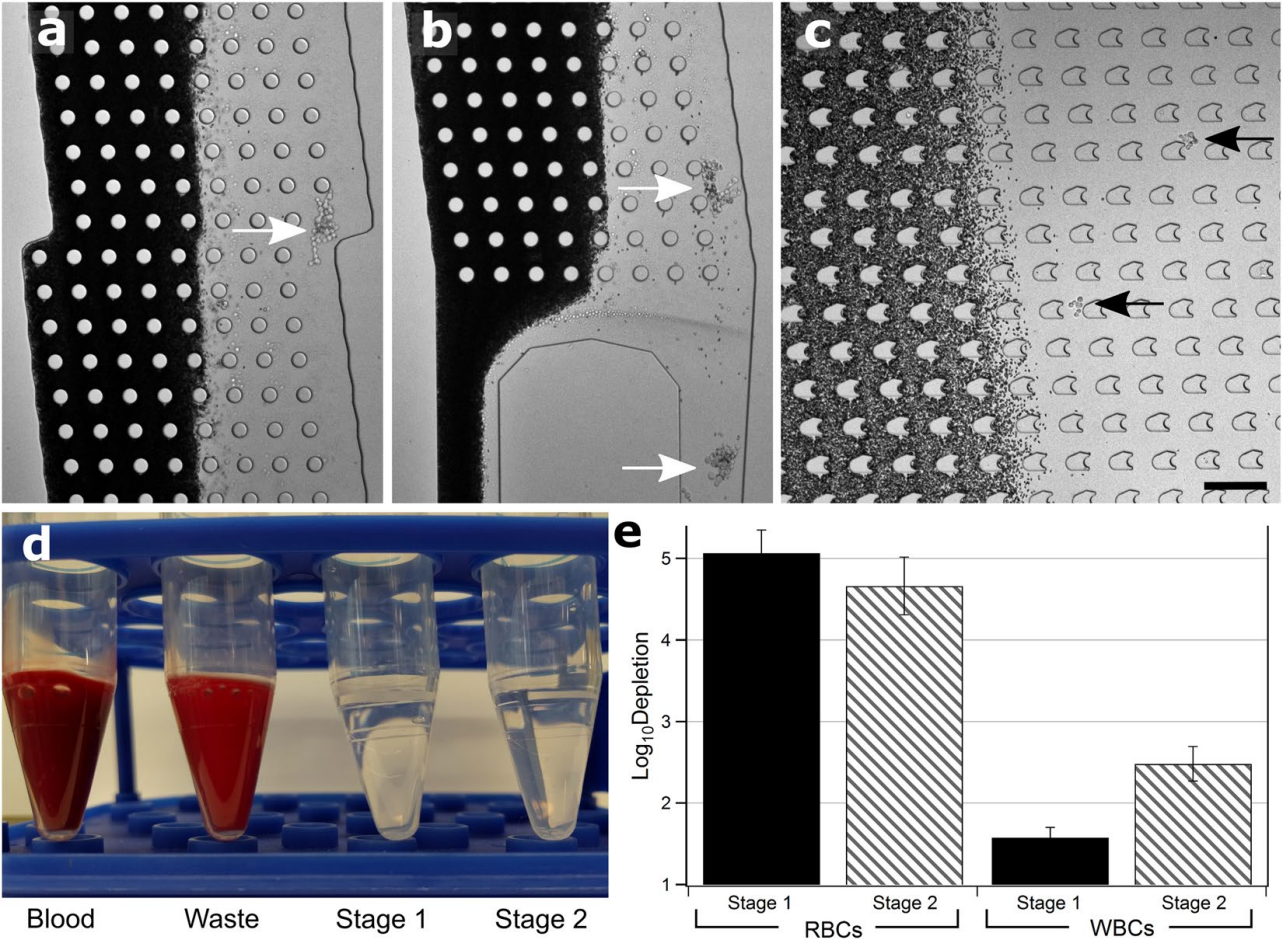
Whole Blood Device Operation. (**a**) Photomicrograph of: mid-Stage 1 showing large cluster deflected into product stream (white arrow), (**b**) exit of Stage 1, two large clusters exiting into Stage 1 product (white arrows), and (**c**) Stage 2 showing two small clusters being deflected out of blood stream (black arrows). Red blood cells are dark. Scale bar: 200 µm. (**d**) Image of Blood input vs. Waste, Stage 1 and Stage 2 outputs showing red blood cell content. (**e**) Log (10) depletion of red blood cells and white blood cells in Stage 1 (dark) and Stage 2 (light) outputs. Experiments conducted in triplicate, error bars represent one standard deviation.

We gave previously reported the *in vitro* culture of CTC clusters from patients with breast cancer. These CTCs proliferate as 3-dimensional clusters in anchorage-impendent culture medium^16^. Suspensions of these cells containing distributions of different sized CTC clusters among single CTCs were spiked into whole blood or buffer before introduction into two-stage cluster capture devices. Figure 2 depicts device operation using cell suspensions in whole blood. In Stage 1, red blood cells remained undeflected while large clusters containing 9 or more cells were effectively deflected (Fig. 2a) into Stage 1 product streams (Fig. 2b and Movie S4). In Stage 2, small clusters were deflected away from blood cells even though their transverse axes were often shorter than the critical diameter of the stage (~30 µm) (Fig. 2c and Movies S5 and S6) suggesting that the asymmetric pillars induced cluster rotation as designed. Both stages were fully integrated into individual devices allowing for simple operation with three inputs (one containing cancer cells/clusters and two containing buffer for co-flow) and three outputs (Stage 1 product, Stage 2 product and Waste) (Fig. 2d). The two-stage DLD cluster capture chip achieved 5.06 ± 0.28 and 4.66 ± 0.35 log_10_ depletion of red blood cells in Stage 1 and Stage 2 products, and 1.58 ± 0.13 and 2.48 ± 0.22 log_10_ depletion of white blood cells in Stage 1 and Stage 2 products, respectively (n = 3) (Fig. 2e). This level of non-cancerous blood cell depletion enabled the direct imaging and analysis of clusters in Stage 1 and Stage 2 products without further processing.

**Figure 3:**
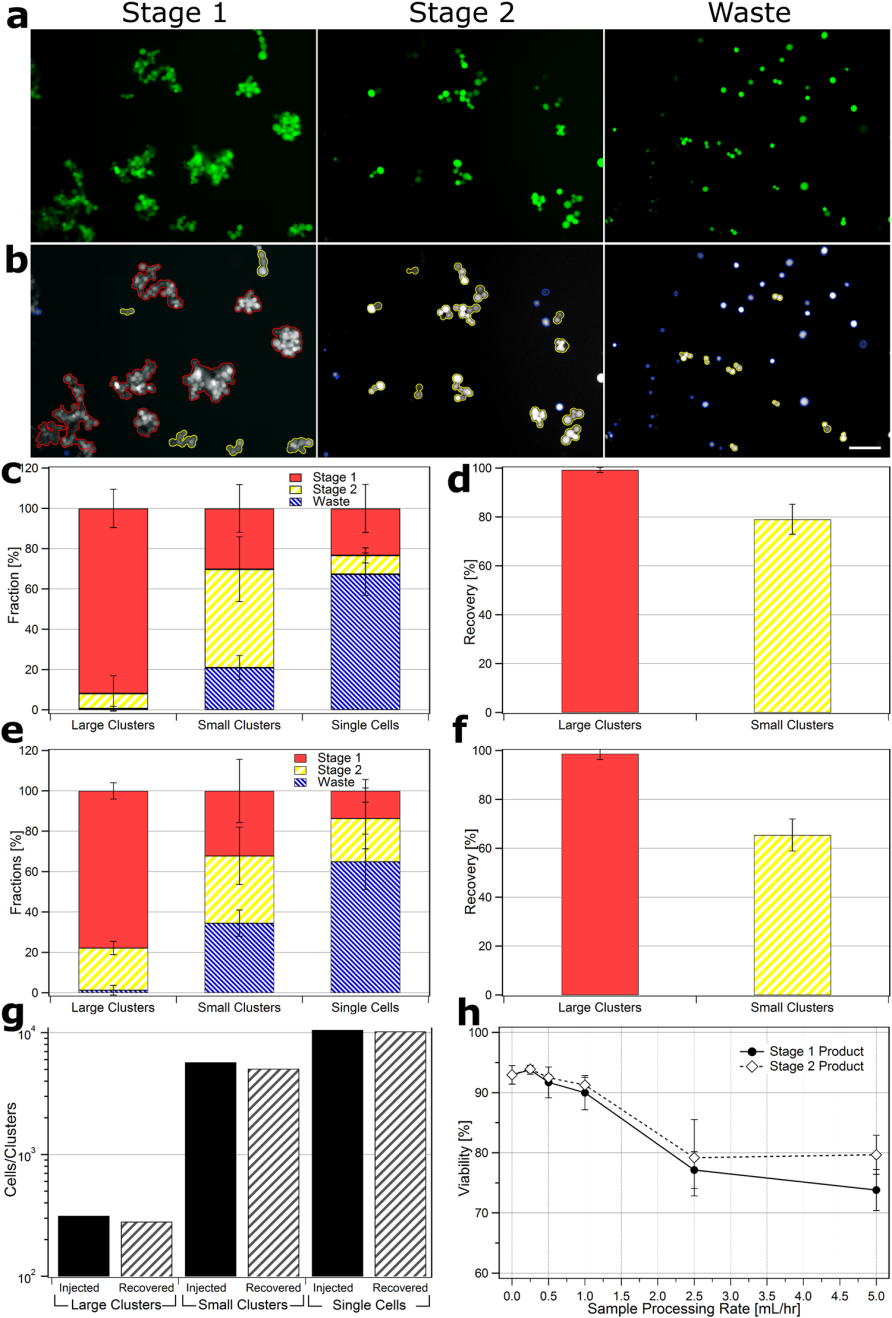
Image Processing and Device Performance. Photomicrographs of frames taken of CTC clusters recovered from whole blood. (**a**) raw unprocessed images and (**b**) processed images of Stage 1 (left), Stage 2 (middle) and Waste (right) outputs with outlines: large clusters (red), small clusters (yellow) and single cells (blue). Scale bar: 100 µm. (**c**) Fraction of total large clusters (red), small clusters (yellow) and single cells (blue) that partition to Stage 1, Stage 2 and Waste outputs for cell/clusters run in buffer. (**d**) Total recovery of large and small clusters into Stage 1 and Stage 2 outputs for runs in buffer. (**e**) Fraction of cells/clusters that partition to outputs for cells/clusters run in whole blood. (**f**) Total recovery of large and small clusters into Stage 1 and Stage 2 outputs for runs in whole blood. Recovery experiments conducted in quintuplicate. (**g**) Comparison of total number of cells/clusters spiked into whole blood before device operation (dark) vs. number collected from all output streams (light). Above experiments all conducted at 0.5 mL/hr blood processing rates. (**h**) Viability of CTCs in Stage 1 product (dark circles) and Stage 2 product (hollow diamonds) 1 hour after operation at different sample processing rates vs. untreated controls (represented by 0 mL/hr). Experiment conducted in duplicate, error bars represent one standard deviation and at least 200 cells were counted per condition.

For analyzing cluster capture efficiencies, a total of 148,945 independent events were analyzed, classified and labeled as either large clusters (red), small clusters (yellow) or single cells (blue) (Fig. 3a,b) (given that clusters sometimes contained dozens of cells the total number of cells analyzed was much greater). Analyses were conducted across 10 independent replicates (five each for clusters run in blood and buffer) using four independent healthy blood donors. Analyses were conducted using custom CellProfiler scripts^17^ which were determined to have greater than 90% accuracy for cell/cluster identification in comparison to manual counting. For analysis, clusters were classified as either ‘Large’ or ‘Small’ using an area cut-off of 3169 µm^2^ (~7500 pixels), which defined small clusters that typically contained eight or fewer cells and large clusters that typically contained nine or greater cells (Fig. S3). Clusters containing well over 100 cells have been successfully isolated from Stage 1 product (data not shown). This cut-off was chosen somewhat arbitrarily for the particular two-stage DLD device geometries. The method used to classify and outline events is summarized in Fig. S4 and an example script is available in the online Supplementary Materials.

Cluster capture efficiencies for inlet blood/buffer flow rates of 0.5 mL/hr are presented in Fig. 3. For CTC cluster cells run in buffer (Figs 3c and S5a), the fraction of isolated large clusters that were recovered in Stage 1 product, Stage 2 product and Waste streams was 91.8 ± 9.5%, 7.5 ± 8.8% and 0.7 ± 1.1%, respectively. The fraction of small clusters in Stage 1 product, Stage 2 product and Waste streams was 30.2 ± 11.8%, 48.9 ± 16.0% and 21.0 ± 6.1%, respectively. Nearly all (99.3 ± 1.1%) large clusters were recovered within the product streams of Stage 1 and Stage 2 products and almost four of every five small clusters were recovered (79.0 ± 6.1%) (Fig. 3d). For CTC clusters run in whole blood (Figs 3e and S5b), the fraction of isolated large clusters that were recovered in Stage 1 product, Stage 2 product and Waste streams was 77.8 ± 4.0%, 20.9 ± 3.3% and 1.3 ± 2.4%, respectively. The fraction of small clusters in Stage 1 product, Stage 2 product and Waste streams was 32.1 ± 15.6%, 33.3 ± 14.2% and 34.5 ± 6.5%, respectively. Similar to runs in buffer, nearly all (98.7 ± 2.4%) large clusters were recovered within the product streams of Stage 1 and Stage 2 products when run with blood (Fig. 3f). However, the total recovery of small clusters was lower (65.5 ± 6.5%). We speculate that this reduced capture efficiency for small clusters may be due to the physical inhibition of cluster rotation in Stage 2 by normal blood cells. Further studies are needed to explore this phenomenon.

### Cluster Stability and Viability

To examine the extent that device operation dissociated clusters into smaller cluster fragments or single cells and to evaluate the total recovery of clusters/single cells, the total number of cells and clusters injected vs. recovered from Stage 1 and Stage 2 outputs of devices operated at 0.5 mL/hr were compared (Fig. 3g). The fraction of injected large clusters, small clusters and single cancer cells that were successfully isolated was 90%, 89% and 97%, respectively, suggesting a high recovery of clusters. Furthermore, since the relative ratios of large clusters, small clusters and single cells remained relatively unchanged after operation, the device appeared to be gentle on clusters, causing minimal shedding of single cells or disruption of clusters into smaller cluster fragments during operation.

To evaluate the detrimental effects of the two-stage cluster chip on cell viabilities, cultured CTC clusters were processed at rates of 0.25, 0.5, 1.0, 2.5 and 5.0 mL/hr and compared to untreated control clusters 1 hour after processing using the trypan blue exclusion assay. The viabilities of cells processed at rates of 0.25, 0.5, 1.0, 2.5 and 5.0 mL/hr (Fig. 3h) were 93.8 ± 0.7%, 91.7 ± 2.5%, 90.0 ± 2.8%, 77.2 ± 3.0% and 73.8 ± 3.4% for Stage 1 products and 93.9 ± 0.6%, 92.5 ± 0.5%, 91.2 ± 1.2%, 79.2 ± 6.4% and 79.7 ± 3.2% for Stage 2 products, respectively, versus control cells with 93.0 ± 1.5%. The viabilities of cells processed at rates of 1.0 mL/hr or less showed no statistically significant difference to control cells (p > 0.05, 95% CI) while those processed at 2.5 and 5.0 mL/hr were significantly different for both Stage 1 and Stage 2 products (p < 0.05, 95% CI). To evaluate the extent of apoptosis in CTC clusters, multispectral flow cytometry was conducted on Stage 1 and Stage 2 products (Fig. S7). The fraction of viable (Calcein-AM positive and Caspase-3/7 negative) cells in Stage 1 output, Stage 2 output, unprocessed spiked into PBS and unprocessed spiked into blood conditions were 87%, 93%, 91% and 97%, respectively suggesting that on-chip processing had minimal effect on the viability of CTC clusters.

To determine if processing hindered the abilities of CTC clusters to proliferate, Stage 1 and Stage 2 products run in two-stage DLD devices at 0.5 mL/hr were collected and cultured in comparison to control untreated clusters. The total number of cells after 5 days were statistically indistinguishable in all cases (Fig. S8) suggesting that the two-stage DLD cluster capture device is an effective tool for isolating CTC clusters for the generation of CTC cell lines and other applications.

## Discussion

There is mounting evidence that circulating tumor cell clusters play a far greater role in the formation of metastatic tumors than previously appreciated^3–5, 18^ and that clusters may be valuable markers for improving our predictive and prognostic abilities in clinical settings^4, 6^. However, our ability to investigate the biology and clinical utility of CTC clusters is hampered by the limited tools capable of reliably isolating and recovering viable clusters from whole blood. The continuous two-stage microfluidic strategy outlined here may remedy this.

Existing strategies for isolating single circulating tumor cells (CTCs) from blood that sort cells based on the expression of cancer-specific surface markers (positive selection), selective removal of non-tumor blood cells (negative selection) or that rely on differences in cell densities, surface characteristics, among others^1, 2^ are effective at sorting cancer cells from noncancerous blood cells, but are poorly equipped to discriminate CTC clusters from individual CTCs since these have similar biochemical and biophysical features. In contrast, this next generation device sorts CTC clusters by interrogating size and asymmetry, two features that in combination result in a potent method of sorting clusters from individual cells in blood. Size-based sorting^1, 2, 19^ used in Stage 1 is effective for capturing large clusters, but when used alone is ineffective for isolating most CTC clusters for a number of reasons: (a) the sizes of individual CTCs vary dramatically (one group reported CTCs with diameters ranging from 4–30 µm within a single patient^20^), (b) the majority of clusters exist as small 2–4 cell aggregates^7^ and (c) asymmetrical particles often assume alignments that “mask” their longitudinal (longest) axes during microfluidic^12, 13^ and filtration^21^ size-based separations. Therefore, a second stage was incorporated to capture clusters that evaded sorting in the first stage by capitalizing on the asymmetry of multicomponent aggregates such as CTC clusters. Asymmetry is an inherent feature in the vast majority of clusters because the arrangement of cells into symmetric configurations is entropically unfavorable (i.e. there are many more ways for cells to organize into asymmetric than symmetric arrangements)^22^, a phenomenon that has been verified in patient CTC clusters^3, 4, 7^.

Altogether, the next generation cluster chip’s two-stage capture strategy is a novel method of isolating viable intact CTC clusters when processing whole blood at rates up to 1.0 mL/hr. The ability to isolate clusters of both large and small sizes is vitally important since the number of cells within CTC clusters isolated from patient varies greatly, from two to over 100 cells^7, 11, 23^. Isolated clusters retained comparable proliferative abilities and levels of apoptosis to untreated controls. Interestingly, clusters isolated in Stage 1 had slightly slower growth rates, viabilities and greater levels of caspase activation than those isolated in Stage 2. More experiments are needed to determine if this is a result of processing or the fact that large CTC clusters, enriched for in Stage 1, have inherently different rates of growth and viabilities, similar to previous reports in tumor aggregates^24^. Comparisons of the distribution of cluster sizes and single cell numbers injected vs. recovered from devices suggest that clusters experience minimal dissociation into smaller clusters or single cells during processing. These advantages over the previous developed device^7^ are likely because during processing, clusters: (a) experience physiological level or lower shear stresses, (b) have short (~10 s) on-chip residence times and (c) are isolated without a discrete release step. The ability to recover high viability CTC clusters likely offset the slightly lower isolation efficiencies for small clusters achieved by this technology especially since extremely rare CTCs and CTC clusters are often “poised on the verge of apoptosis”^4^ and therefore need to be gently manipulated if they are to be analyzed and cultured. To increase blood processing rates, future device iterations and/or process modifications such as increased array widths, sample pre-concentration and/or parallelization^25^ could be pursued. In addition, future iterations may incorporate DLD arrays with smaller critical diameters to increase the capture efficiency of small clusters if desired. Gains in cluster recovery however, will likely be offset by increased device clogging and/or contamination of large single cells in product streams.

An added benefit of the two-stage capture strategy is that the of sequential isolation of clusters by size and asymmetry results in two outputs which are enriched for small and large clusters respectively. These size enriched outputs may be useful since cluster size has emerged as a potentially important area of investigation that may affect the function, composition and potency of clusters. Large clusters appear to be more heterogeneous^11, 26^, contain more viable cancer cells^26^, form more metastases when injected into animals^27, 28^ and are correlated with worse overall survival rates of cancer patients^29^ than small clusters. However, the mechanisms responsible for these differences have been almost entirely unexplored because of a lack of tools capable of isolating and enriching clusters of different sizes. This device may therefore be a valuable tool for researchers in this area by enabling the isolation and enrichment of different sized clusters on demand so that these mechanisms can be systemically studied.

## Conclusions

The two-stage strategy described here is capable of the high efficiency isolation of viable CTC clusters and is unique in its ability to discriminate clusters based on both their size and inherent asymmetry. This method is advantageous because it is label-free, engineered to operate with low shear stress (physiological or less throughout), is continuous, which reduces on-chip residence times and enables in-line integration with other CTC analysis or capture technologies, and sorts large and small clusters into different output streams. The isolation of viable CTC clusters from blood has the potential to enable significant advances in our understanding and treatment of metastatic cancer.

## Materials and Methods

### Device Fabrication and Preparation

Devices were fabricated using standard soft lithography techniques. Briefly, two-layer masters were fabricated by spin coating SU-8 negative photoresist (Microchem, Newton, MA) onto silicon wafers. Coated wafers were patterned using UV photolithography to define masters for devices (Fig. 1a) consisting of 90 µm high ‘Stage 1’ DLD sections (Fig. 1b,c) that consisted of 50 µm diameter cylindrical micropillars with 63 µm lateral gaps, 16 µm shifts and row shift fractions of 1/7 for nominal critical diameters of 30 µm, arrayed into 8 columns and 10 rows per reset with 12 total resets. Stage 1 had two fluidic inlets and one fluidic outlet. The deflected stream of Stage 1 was coupled with 340 mm long, 200 µm high channel which compensated for the resistance of the undeflected product to provide a 1/3 fraction of fluid for ‘Stage 1 product’. When necessary to compensate for high blood hematocrit values, this output was coupled to an additional a 20–60 cm long segment of 0.25 mm inner diameter TYGON® flexible plastic tubing (Cole-Parmer, Vernon Hills, IL). The undeflected product of Stage 1 was fed into 30 µm high ‘Stage 2’ DLD sections (Fig. 1d,e) that consisted of 77 × 60 µm (width × height) custom micropillars which were half “I”-shaped and half ellipsoidal, designed to induce X-Y planar rotation of clusters (Fig. 1g). Stage 2 micropillars are spaced with 63 µm lateral gaps, 20 µm shifts and row shift fractions of 1/7 for approximate nominal critical diameters of 30 µm, arrayed into 32 columns and 10 rows per reset with 48 total resets. Stage 2 had one fluidic buffer inlet in addition to the Stage 1 inlet and two fluidic outlets. One third of the total flow of Stage 2 was deflected as ‘Stage 2 product’. The remaining undeflected output was considered ‘Waste’. For device development and characterization of asymmetric pillars, devices that consisted of DLD arrays featuring standard cylindrical or asymmetric pillars with 30 or 90 µm high ceilings with nominal critical diameters of 30 µm and gap/row shifts as described above were used. The relative lengths of outlet channels were varied accordingly to maintain fluidic splits by matching the ratios of outlet resistances to those described above. The heights of SU-8 features were verified to be within ± 10% of setpoints using a surface profilometer (Dektak ST System Profilometer, Veeco Instruments Inc., Plainview, NY). Polydimethylsiloxane (PDMS) prepolymer mixed with its crosslinker (Dow Corning, Midland, MI) at 10∶1 ratio (w/w) was poured onto silicon master molds, degassed, and cured at 65 °C for 24 h. Cured PDMS replicas were removed from the molds and punched using 1.2 mm or 1.0 mm Harris Uni-Core™ biopsy punchers. Punched PDMS slabs were oxygen plasma treated at 300 mmTor O_2_ at 50 W for 35 s, and bonded to glass slides to form microfluidic devices. Devices were primed with 300 µL 70% (v/v) ethanol in water, flushed with 300 µL phosphate buffered saline (PBS), blocked with 3% Pluronic F68 (w∕v) (ThermoFischer Scientific, Waltham, MA) in PBS for 45 minutes at room temperature and then flushed with 300 µL PBS. 40–50 cm and 5–10 cm long sections of TYGON® flexible plastic tubing with an inner diameter of 0.50 mm were then coupled to fluidic inlets and outlets, respectively, as necessary.

### CTC Cluster Culture and Preparation

Reagents for cell isolation, culture and preparation were obtained from Life Technologies (Carlsbad, CA) unless noted otherwise. *Ex-vivo* cultured breast cancer CTC clusters isolated from two patients (50 and 142) were cultured as previously described^16^. Briefly, CTC and CTC clusters were isolated from blood biopsies of metastatic breast cancer patients using antibody affinity capture^30^. CTC clusters engineered to express GFP and luciferase^16^ were used for most experiments while untagged parental CTC clusters were used for viability experiments that required staining. Cultured CTC-clusters were grown as multicellular clusters in suspension in ultralow attachment 6 well plates (Corning Inc., Corning, NY) in RPMI-1640 media supplemented with 20 ng/mL epidermal growth factor, 20 ng/mL, basic fibroblast growth factor, 1X B-27® supplement and 1X Antibiotic-Antimycotic at 37 °C, 5% CO_2_ and 4% O_2_. Media was exchanged every 4–5 days.

Suspensions containing mixed populations of cultured individual CTCs and CTC clusters were prepared by collecting and applying gentle mechanical dissociation to 2 mL cultured CTCs collected directly before media exchange at concentrations of 0.25–1.25 × 10^6^ cells/mL. Cell suspensions were then centrifuged at 200× g for 5 minutes and fractions of the pellet resuspended in 2 mL of healthy donor blood collected in ACD or EDTA BD Vacutainer™ blood collection tubes (BD Biosciences, Franklin Lakes, NJ) or PBS to final concentrations of 1.0 × 10^3^–1.0 × 10^6^ cells/mL. Blood specimens were obtained from healthy donors after informed consent and according to approved experimental protocols per the Partners Human Research Committee Institutional Review Board protocol #2009-P-000295 at Massachusetts General Hospital. For nuclear visualization, cell solutions were stained by introducing Hoescht 33342 to a final concentration of 16.2 µM for 10 minutes. For viability determination, 10 µL samples of well-mixed cluster containing solutions (untreated controls vs. devices products) were added to 10 µL of 0.4% trypan blue solution (Sigma Aldrich, St. Louis, MO) and mixed rigorously before injection and counting on a haemocytometer (Hausser Scientific, Horsham, PA). At least 200 cells were counted per condition.

To compare the growth rate of clusters, untreated control clusters and clusters from Stage 1 and Stage 2 products collected after isolation in two-stage DLD devices (as described below) were haemocytometer counted (as described above) and seeded into ultra-low attachment 96 well plates (Corning, Inc.) at 10,000 viable cells per well in 100 µL complete media. A total of nine wells per condition were generated, and plates were incubated at 37 °C, 5% CO_2_ and 4% O_2._ On days 1, 3 and 5, three wells in each condition were collected and their contents mixed with trypan blue and haemocytometer counted.

### Device Operation

Three syringe pumps (Harvard Apparatus, Holliston, MA) were used to introduce solutions into devices. Two pumps were loaded with 10 mL syringes (BD Biosciences) containing 10 mL PBS. The remaining pump was loaded with a 3 mL syringe containing CTC blood/cell suspensions (prepared as described above) and all pumps were coupled with appropriate device inlets. For the majority of experiments and unless otherwise specified, syringe pumps were driven at flow rates of 8.3 µL/min (0.5 mL/hr), 31.6 µL/min and 40 µL/min into cluster blood/cell inlets, Stage 1 buffer inlets and stage 2 buffer inlets, respectively, via TYGON™ tubing. For viability experiments, syringe pumps were driven at 0.5, 2.0, 5.0 and 10.0 times these rates (maintaining relative ratios of flow rates) to generate sample flow rates of 0.25, 1.0, 2.5 and 5.0 mL/hr, respectively. Stage 1 product, Stage 2 product and Waste outputs were fed into separate wells of ultra-low attachment 24 well plates (Corning, Inc.). Devices were operated for 1–2 hours before flows discontinued. 10–100 µL samples of each input/output were sampled for blood cell quantification (described below). For the majority of runs which operated in blood, waste solutions were gently mixed with 10 mL 1X red blood cell lysis solution (Miltenyi Biotec, Cambridge, MA) for 15–20 minutes at room temperature followed by a centrifugation at 200 × g for 5 minutes and the pellet resuspended in 1.5 mL PBS and reintroduced into an unused well of the ultra-low attachment well plate. In control experiments, this process was not found to disrupt clusters. The contents of the wells were then imaged by recording 330 frames/well using an inverted fluorescence microscope with automated stage (Eclipse 90i, Nikon, Melville, NY) under a 10X objective. These frames provided nearly full surface coverage in each well. High speed videos were recorded using a Phantom v4.2 camera (Vision Research, Wayne, NJ).

### Automated Cell Enumeration

Recorded images of GFP tagged cells and clusters in wells plates collected as described above, were imaged using a custom CellProfiler (version 2.1.1 rev 6c2d896)^17^ script designed to rapidly enumerate the number of single cells, ‘small clusters’ and ‘large clusters’ in each frame. The script (an example processing outlined in Fig. S4.) (i) uploaded raw fluorescent images; (ii) identified all cells, both singles and within clusters using adaptive background thresholds; (iii) identified the number of cell ‘neighbors’ (cells with membranes in direct contact); (iv) classified singles cells as those without any neighbors; (v) identified clusters as groups of cells with neighbors; (vi) classified ‘large clusters’ as those with areas greater than 3169 µm^2^ (corresponding to ~8.67 cells); (vii) classified the remaining clusters as ‘small clusters’; and (viii) overlaid outlines for single cells (blue), small clusters (yellow) and large clusters (red) onto 8-bit converted raw images. Image outputs were manually checked for image processing errors. Background thresholding and declumping values were tailored to each experiment to compensate for differences in fluorescent and background intensities. An example script is available for download in the Supplementary Materials.

For experiments comparing the number and distribution of cells and clusters introduced into versus recovered from devices, mixed population CTC cell/cluster suspensions were collected before media exchange (as described above), and introduced into ultralow attachment well plates. Image analyses were conducted using automated well scanning (as described above). These suspensions were then spiked into whole blood, processed on chip, recovered from all three outputs and the cells/clusters enumerated as described above.

### Imaging flow cytometry for viability and apoptosis

The viability and degree of apoptosis of CTC clusters in Stage 1 product, Stage 2 product, unprocessed spiked clusters in PBS and unprocessed spiked clusters in whole blood, were analyzed using the ImageStream Mark II multispectral imaging flow cytometer (Amnis Corp., Seattle, WA) using a 40x objective and 405 nm, 488 nm, and 642 lasers. For cells recovered in output streams or spiked into PBS, solutions were centrifuged at 300 × g for 4 mins, resuspended in RPMI media containing 10 mM HEPES buffer and 0.3% BSA, and stained with DRAQ5 (1 µM; Cell Signaling Technology, Danvers, MA), Calcein Blue AM (2.5 µM; Life Technologies), CellEvent Caspase-3/7 Green Detection Reagent (2 µM; Life Technologies), PE-conjugated EpCAM antibody (1:286; clone VU1D9; Cell Signaling Technology), and PE-CF594-conjugated CD45 antibody (1:400; clone HI30; BD Biosciences). For analyzing spiked cells in whole blood, solutions were diluted 33-fold in RPMI media containing 10 mM HEPES and stained with DRAQ5 (1 µM), Calcein Blue AM (6.25 µM), CellEvent Caspase-3/7 Green Detection Reagent (4 µM), PE-conjugated EpCAM antibody (1:500), and PE-CF594-conjugated CD45 antibody (1:400). Nucleated cells were gated using DRAQ5 and spiked cancer cells were identified as EpCAM-positive and CD45-negative. At least 140 individual cells were manually classified per condition, cells were considered viable if they stained positive for Calcein Blue AM but negative for Caspase-3/7 and non-viable if they stained positive for Caspase-3/7 or negative for Calcein Blue AM.

### Quantifying Blood Cell Depletion

The concentration of red blood cells (RBCs) in input solutions spiked with CTCs (as described above) and in output solutions after experiments were determined by analyzing 100 µL samples of these solutions, collected as described above, in a KX-21N Hematology-Analyzer (Sysmex, Linconshire, IL). For Stage 1 and Stage 2 products, the concentration of RBCs was determined by manual counting 10 µL samples of these solutions on a haemocytometer.

The concentration of white blood cells (WBCs) in Stage 1, Stage 2 and Waste solutions were determined by staining a solution of CTCs spiked into whole blood with Hoescht 33342 prior to processing on chip. Twenty randomly assigned frames per condition were taken in both GFP and Hoescht 33342 channels and manually counted. The number of cancer cells (GFP) were subtracted from the total nuclei (Hoescht 33342) to determine the number of white blood cells per condition. Total RBC and WBC numbers were determined by multiplying total input/output volumes by concentrations for comparison.

### Computational Fluid Dynamics Model

A full-width steady-flow model of each DLD stage was performed in three dimensions to predict flow profiles and estimate the wall shear stresses experienced in the integrated device. In each case, pressure inlet and outlet boundary conditions were applied such that the design flow rate was achieved, and a uniform viscosity of 1 mPa-sec was assumed for the working fluid. Although in practice blood viscosity is non-Newtonian and typically has a higher effective viscosity, this greater viscosity also reduces the velocity of blood in relation to buffer co-flows thereby reducing the magnitude shear stress. This compensating blood viscosity effect makes a uniform buffer viscosity a reasonable assumption for estimating the magnitude of shear stress experienced at walls. To visualize the flow, streamlines were generated along the mid-plane of the channel and wall shear stress was plotted on the rear walls (with front walls suppressed to enable visualization; color scale: 0 to 7 Pa). The task of meshing and problem solution were each performed with a commercial software package (ANSYS Fluent, version 13). Stage 1 DLD module repeating units were composed of smooth circular posts and a 90-μm-thick mesh was constructed by projecting a 2D surface triangular mesh on the channel roof to the floor. This resulted in 1.98 × 10^6^ prism elements distributed in thirty 3-μm-thick layers. Stage 2 DLD module repeating units were composed of complex shaped posts and the 30-μm-thick mesh was constructed of 8.48 × 10^6^ tetrahedral elements.

## Electronic supplementary material

Movie S1

Movie S2

Movie S3

Movie S4

Movie S5

Movie S6

Supplementary Info

Supplementary Info
