# Supplementary material for: Microfluidic Isolation of Circulating Tumor Cell Clusters by Size and Asymmetry
Source: Sci Rep. 2017 May 26;7:2433. doi: 10.1038/s41598-017-01150-3 (PMC5446400; doi:10.1038/s41598-017-01150-3)
Supplement: Supplementary file 7 — Supplementary Info [file 41598_2017_1150_MOESM7_ESM.pdf]

Supplementary Information for Microfluidic Isolation of Circulating Tumor Cell Clusters by Size and Asymmetry

Sam H. Au[1,2], Jon Edd[1], Amy E. Stoddard[1], Keith H.K. Wong[1,2], Fabio Fachin[1], Shyamala Maheswaran[2,3], Daniel A. Haber[3,4,5], Shannon L. Stott[1,3,4], Ravi Kapur[1] and Mehmet Toner[1,2,6*]

[1] Center for Engineering in Medicine, Massachusetts General Hospital, Harvard Medical School, Boston, MA 02114.

[2] Department of Surgery, Massachusetts General Hospital, Harvard Medical School, Boston, MA 02114.

[3] Massachusetts General Hospital Center for Cancer Research, Harvard Medical School, Charlestown, MA 02129.

[4] Department of Medicine, Massachusetts General Hospital, Harvard Medical School, Boston, MA 02114

[5] Howard Hughes Medical Institute, Bethesda, MD, 20815.

[6] Shriners Hospital for Children, Boston, MA, 02114.

Supplementary Figures

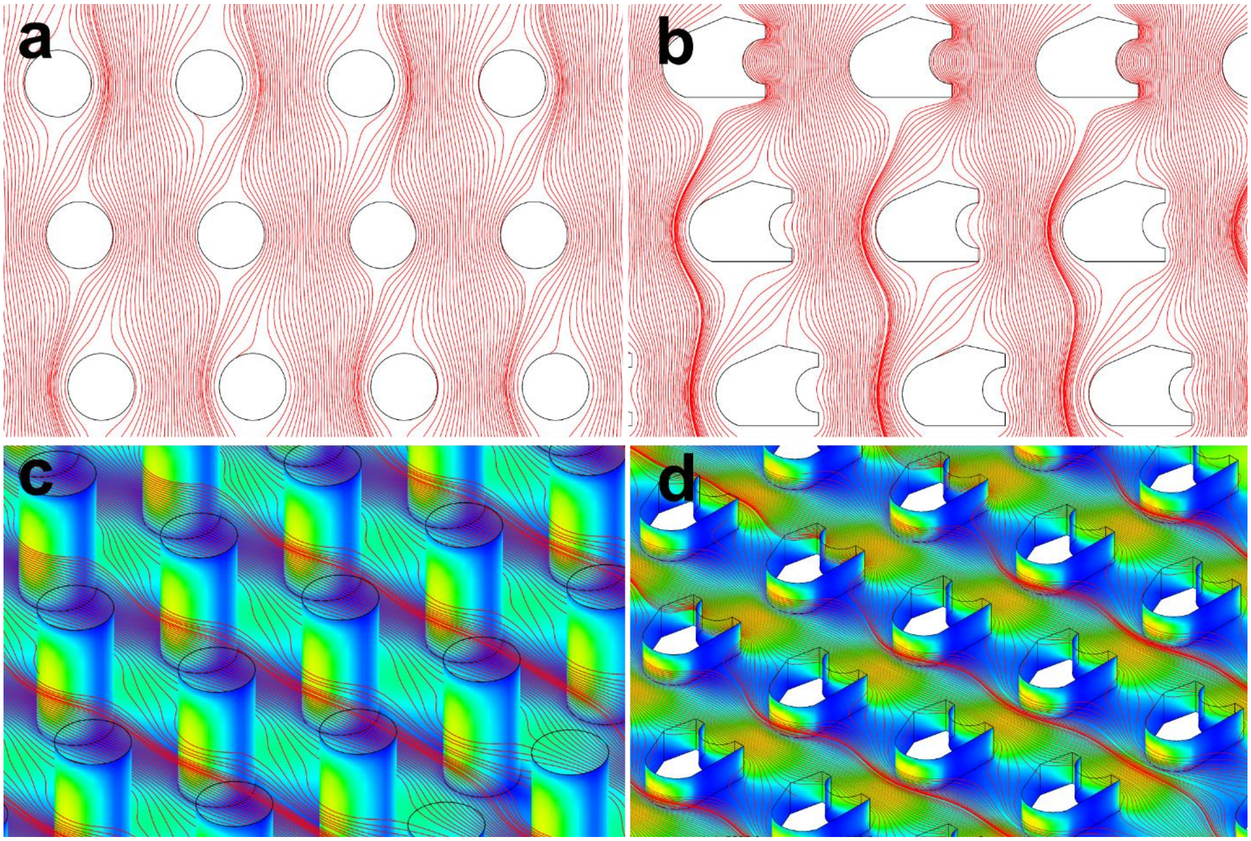

Fig S1: Computation fluid dynamic models. Mid-plane cut plane projections showing (a) Stage 1 – undisturbed fluid streamlines and (b) Stage 2 – streamline asymmetry between pillar. 3D computation model with streamlines (red lines) and shear stress intensity (colored planes) for processing 0.5 mL blood per hour: (c) Stage 1, peak shear stress = 2.9 Pa, (d) Stage 2, peak shear stress = 4.8 Pa. Magnitude of shear stress ranges from 0-7 Pa (green-red).

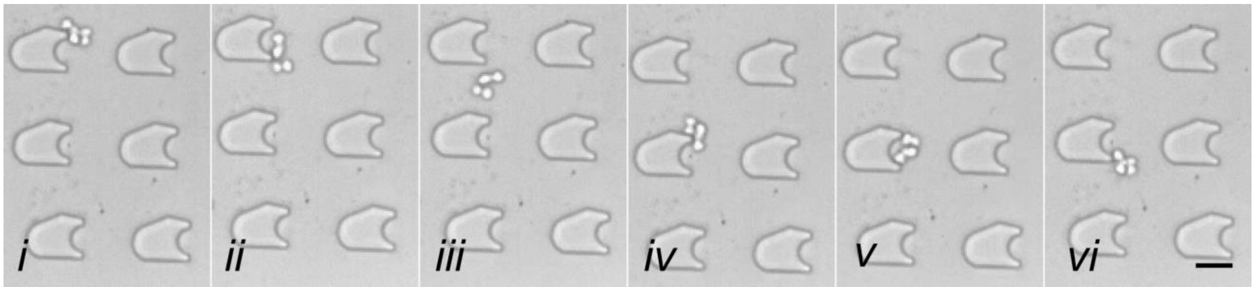

Fig S2: Frames captures using a high speed camera showing rotation of small cluster as it transits past asymmetric pillars in Stage 2. Scale bar: 50 µm

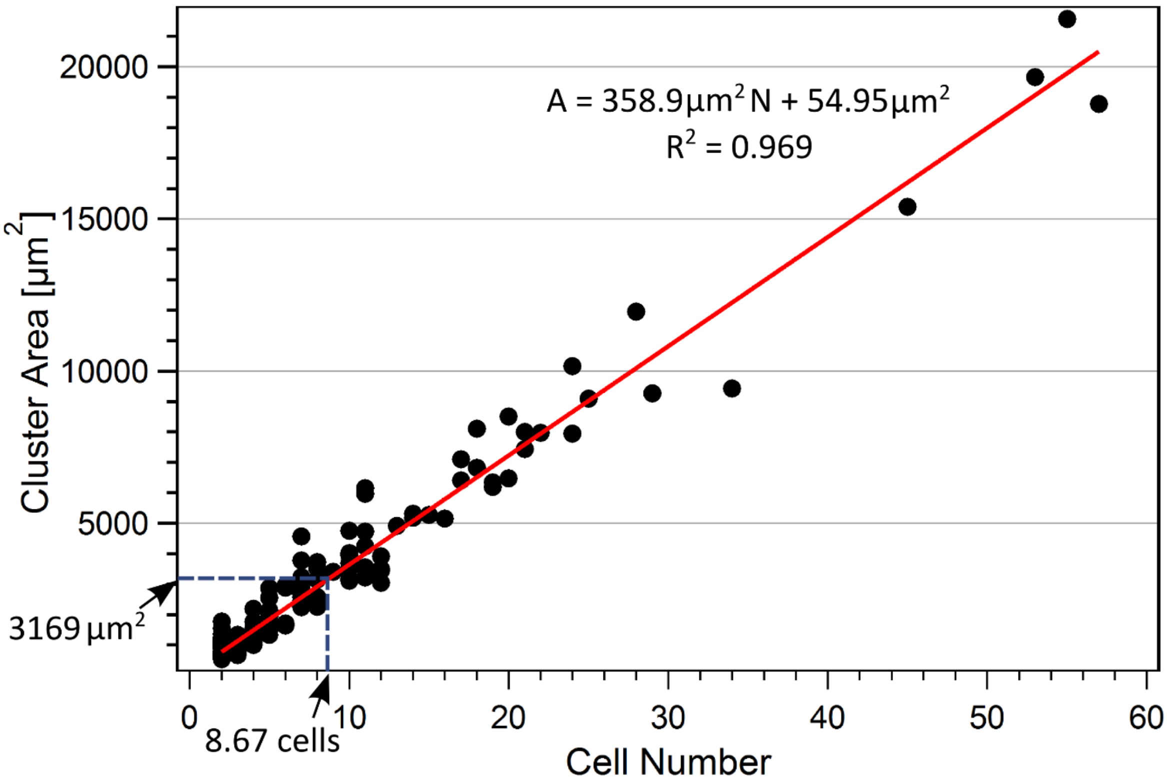

Fig S3: Correlation of area vs. cell number for 203 cluster events analyzed using custom Matlab® scripts. Large/Small cluster cut-off was defined as 3169 μm^2 which corresponded to a cut-off of ~ 8.67 cells. Data fit to least squares linear regression.

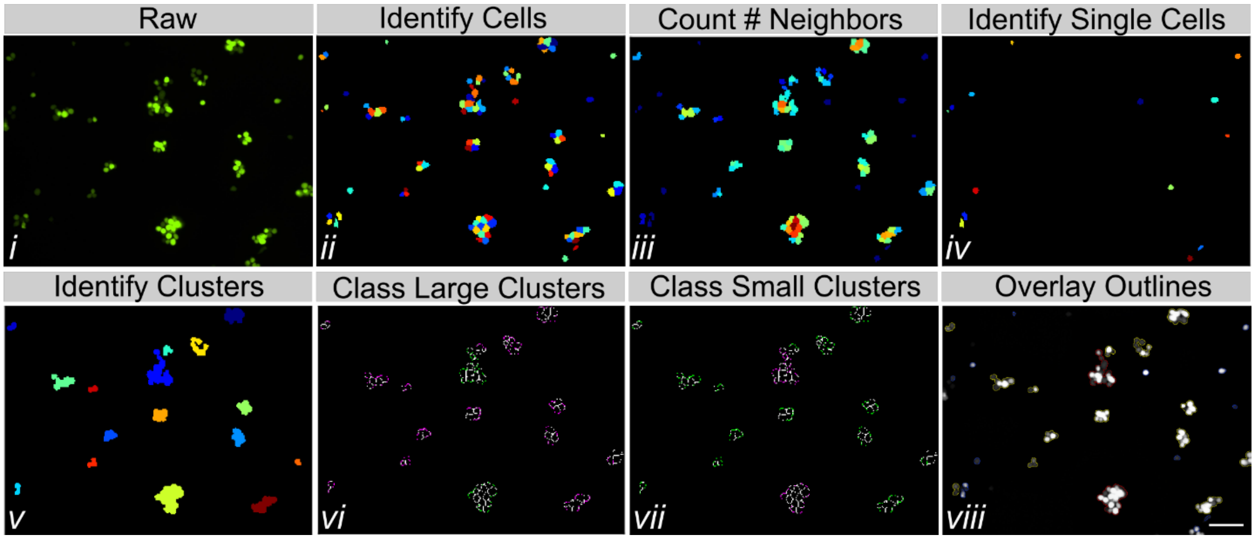

Fig S4: Sample image processing pipeline showing processing of a raw image into final image with overlayed outlines.

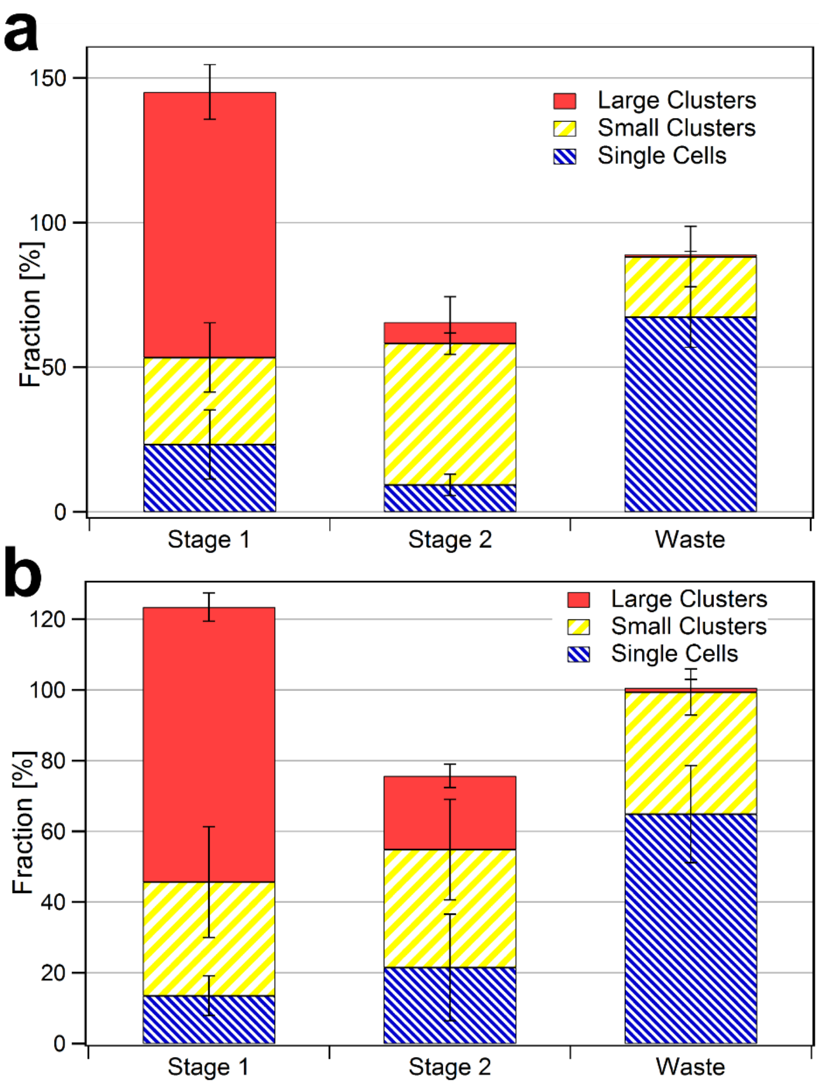

Fig S5: Fraction of total large clusters (red), small clusters (yellow) and single cells (blue) that partitioned to Stage 1 product, Stage 2 product and Waste outputs for (a) cell/clusters run in buffer and (b) cell/clusters run in whole blood. Data is an alternative plotting of figures 4a,c.

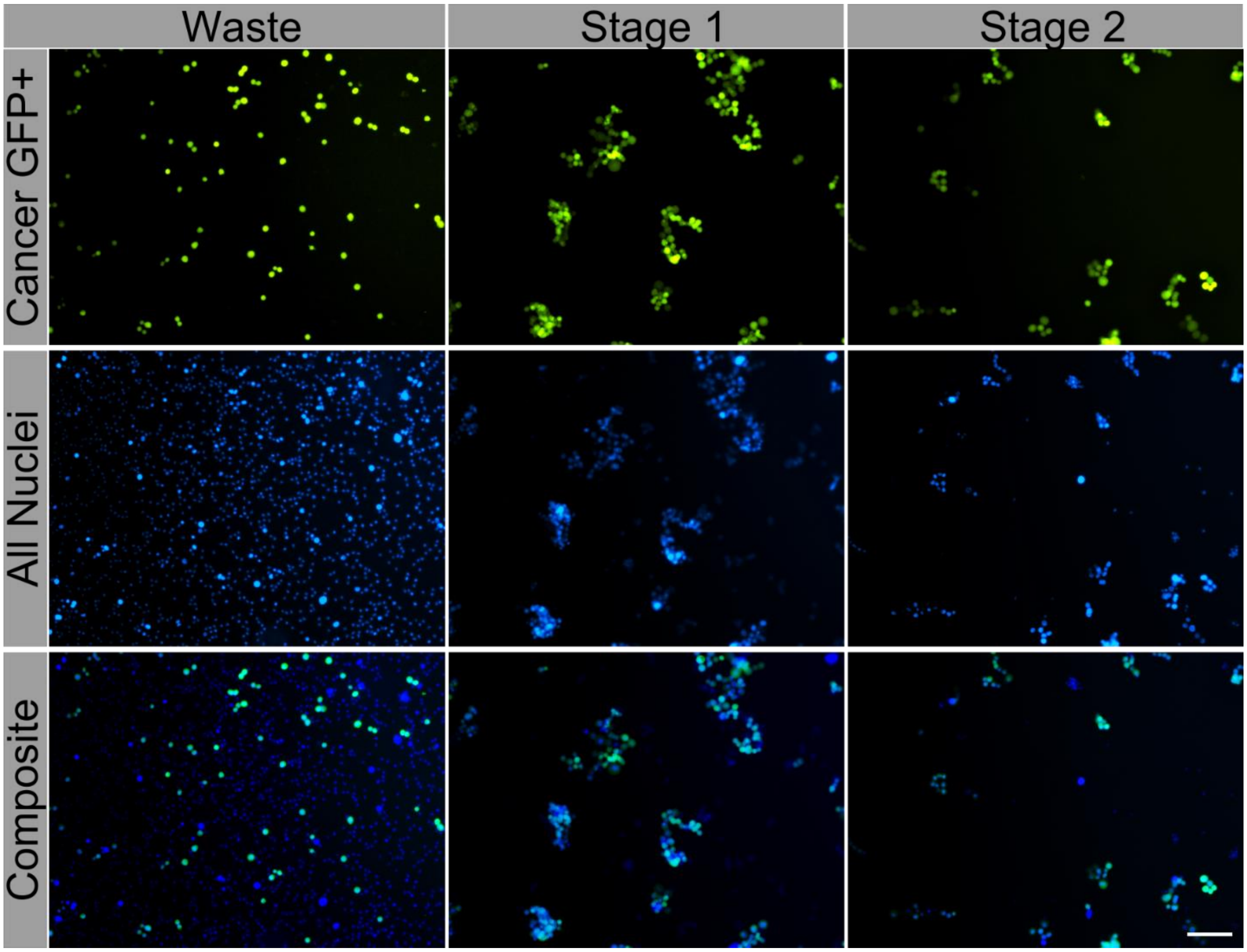

Fig S6: Sample images used for determining white blood content in Waste, Stage 1 and Stage 2 outputs. Cancer cells express GFP (top), nuclei stained with Hoescht 33342 (middle) and composite (bottom). Scale bar: 100 µm.

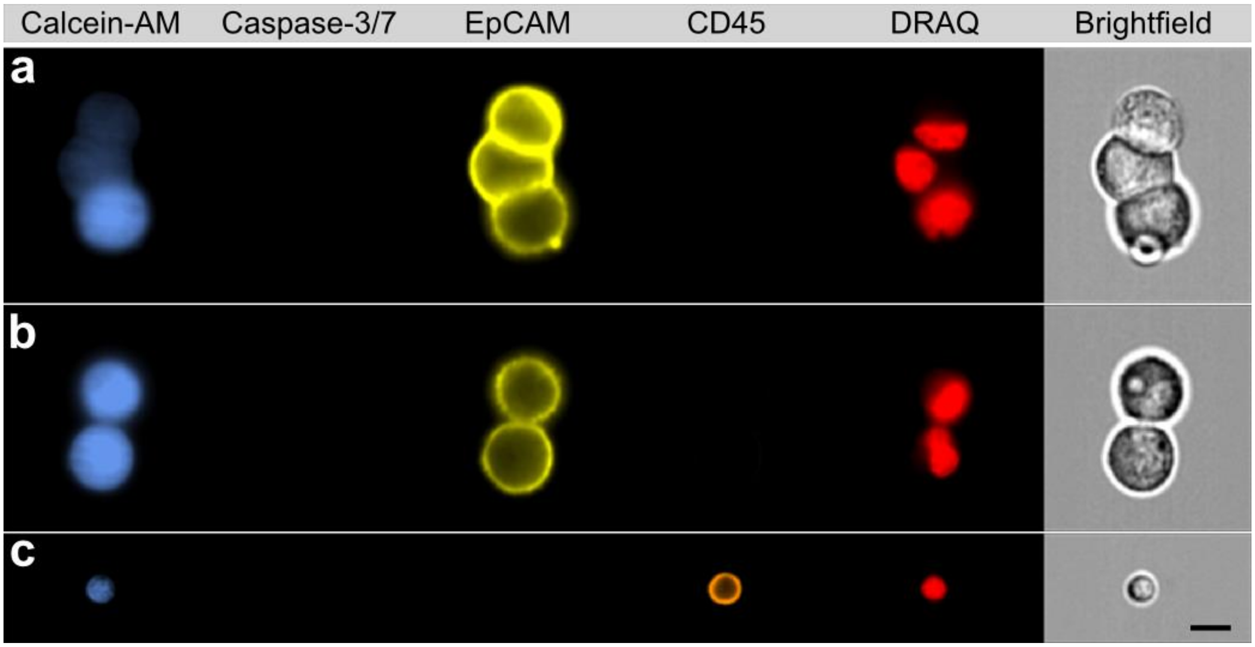

Fig S7: Multispectral flow cytometry images of EpCAM+/CD45- CTC clusters from (a) Stage 1 product and (b) Stage 2 product; with (c) EpCAM-/CD45+ white blood cell from Stage 1 product for comparison. Cells were stained for viability (blue), Caspase-3/7 (green), EpCAM (yellow), CD45 (orange) and DNA (red). Scale bar: 10 µm.

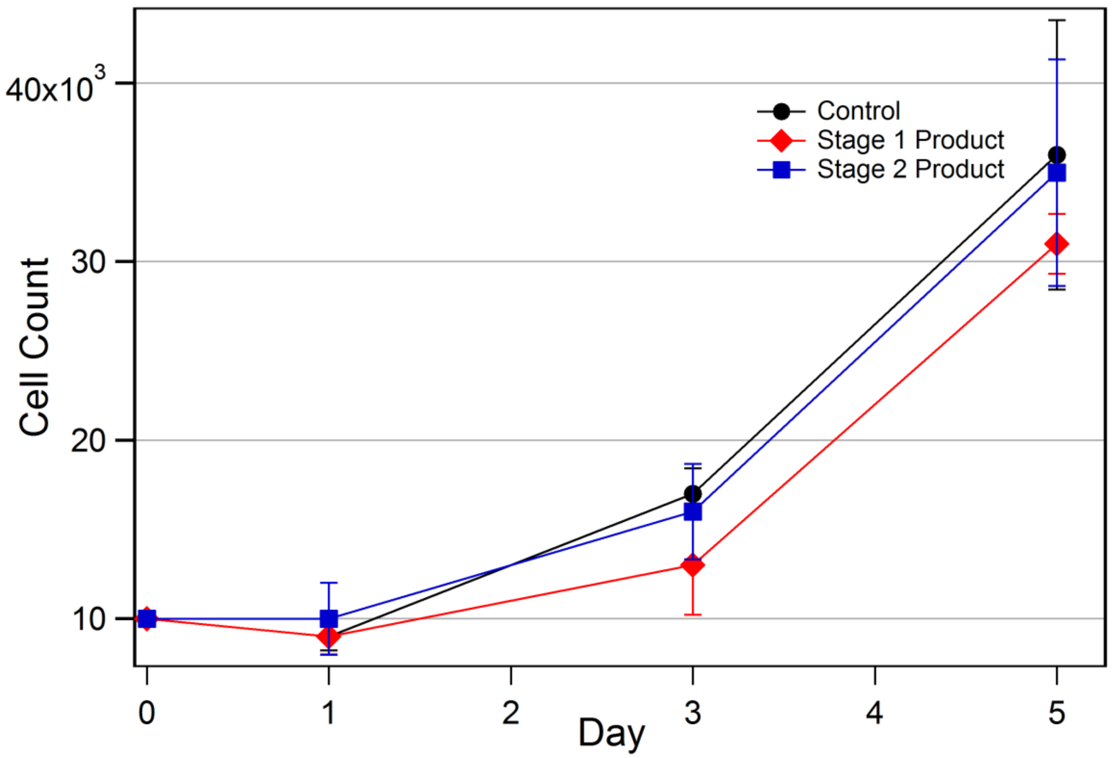

Fig S8: Growth curve of CTC clusters cultured for five days after isolation in ultra low attachment 96 well plates: control (black circles), Stage 1 product (red diamonds) and Stage 2 product (blue squares). Error bars represent one standard deviation, experiment conducted in triplicate.

Movies

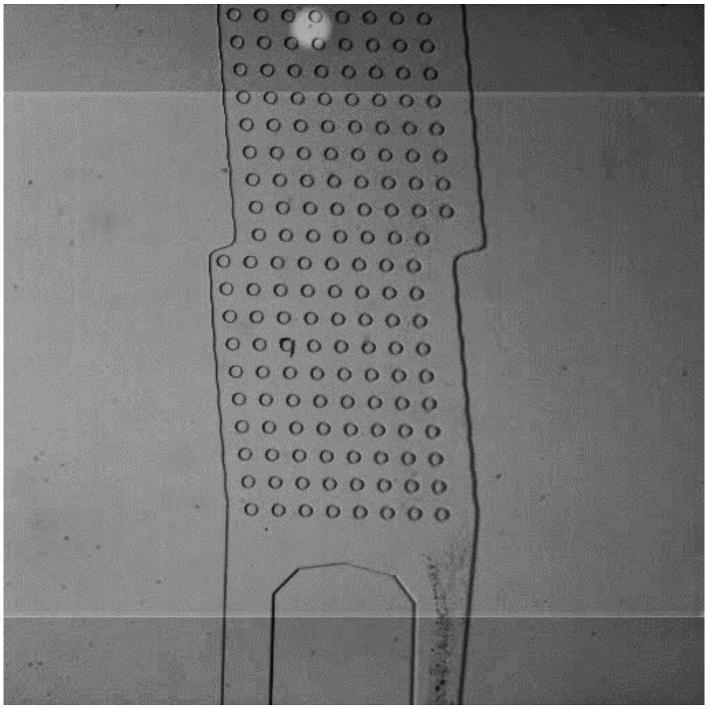

Movie S1: High speed video of two-cell cluster tending to align along vertical axis during sorting through array of cylindrical pillars in 90 µm high Stage 1 device.

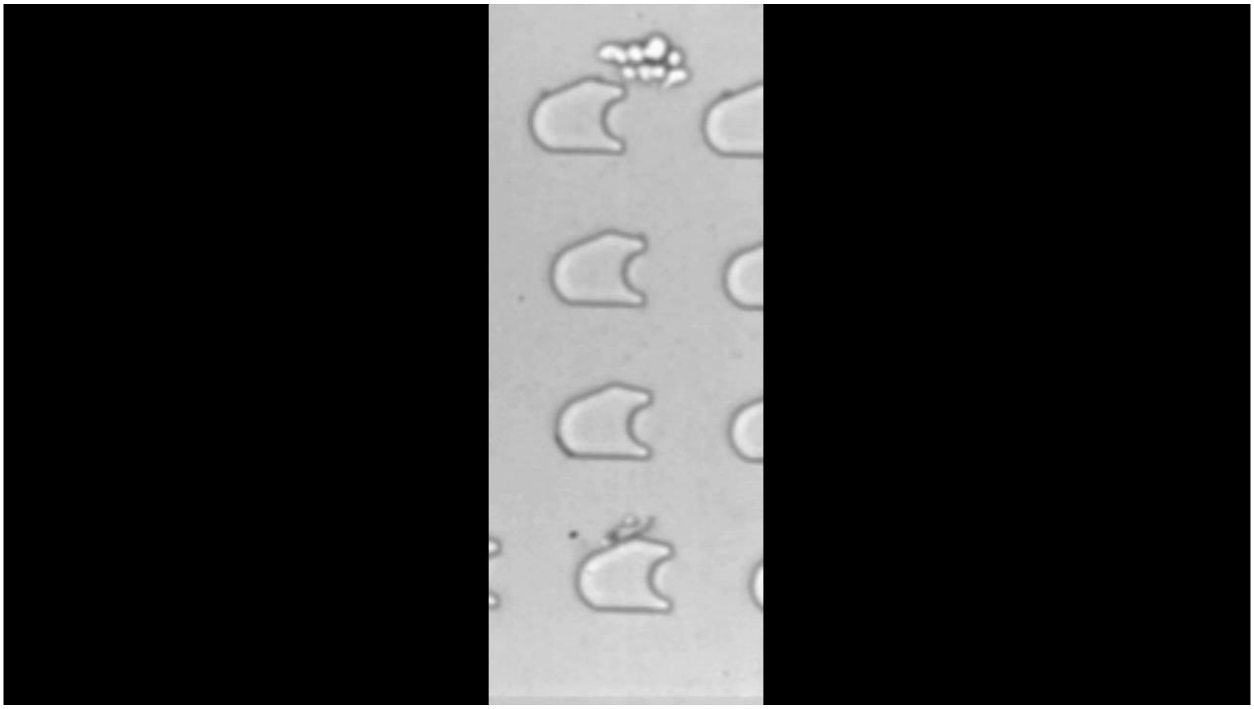

Movie S2: High speed video capturing small CTC cluster behavior. Custom designed pillars in 30 µm high device effectively deflect small CTC cluster because of cluster rotation.

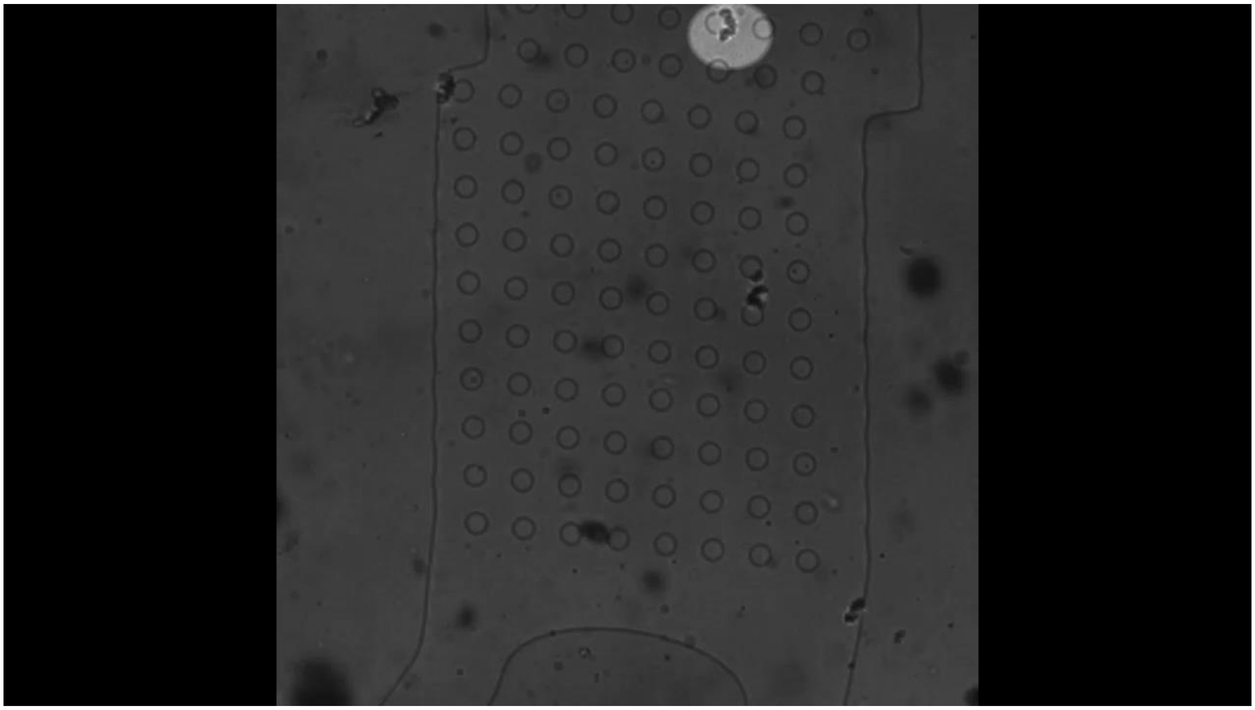

Movie S3: Cylindrical pillars in 30 μm high device is unable to deflect small CTC cluster because of minimal rotation behavior. Cluster ends up in Waste (left) stream.

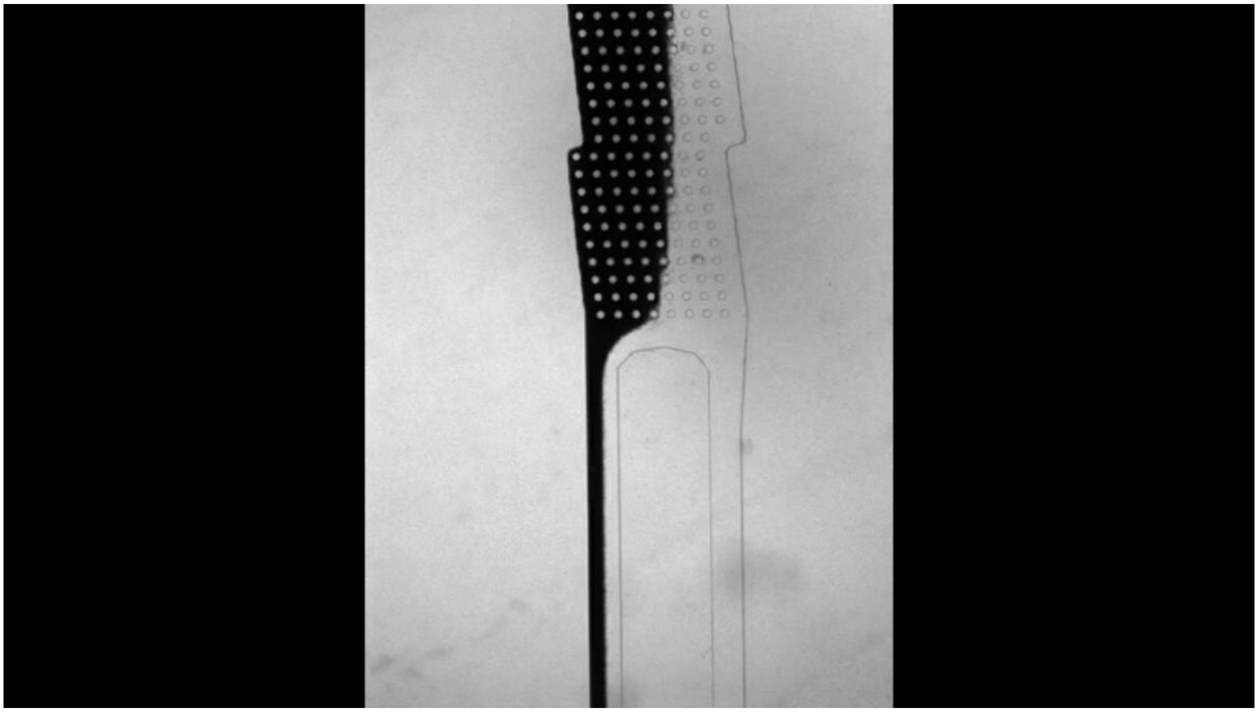

Movie S4: Exit streams of Stage 1 showing deflection of CTC clusters out of whole blood in 90 μm high device.

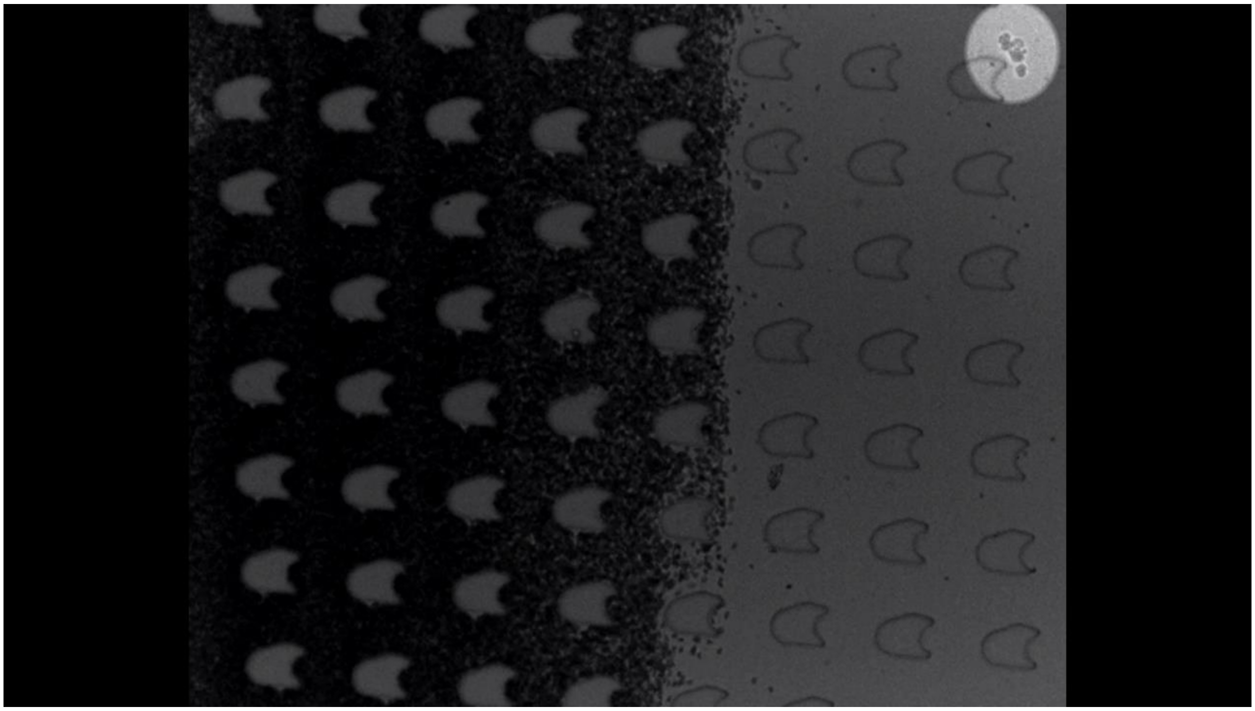

Movie S5: Two sequential CTC clusters being rotated and bumped by asymmetric pillars allowing deflecting even though transverse axis are less than critical diameters in Stage 2 pillars in 30 µm high device.

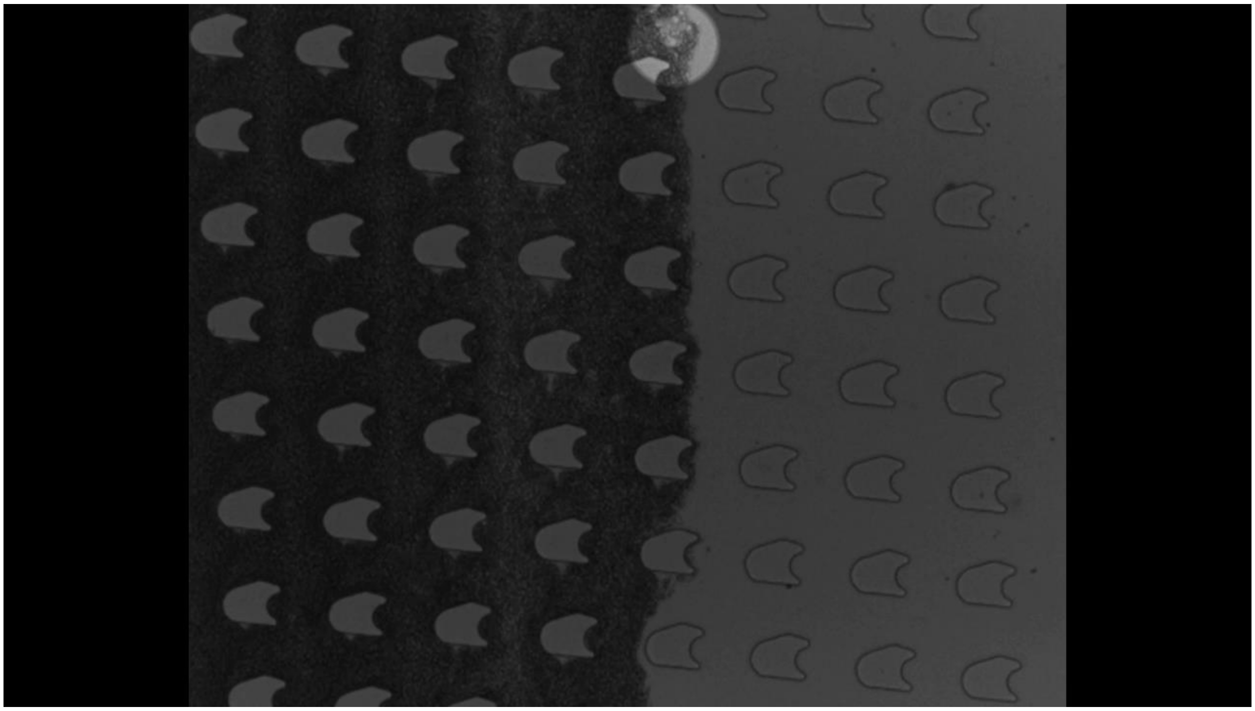

Movie S6: CTC clusters being deflected out of whole blood by Stage 2 pillars in 30 µm high device.
